# Validated assessment tools for screen media use: A systematic review

**DOI:** 10.1371/journal.pone.0283714

**Published:** 2023-04-13

**Authors:** Oriana Perez, Tatyana Garza, Olivia Hindera, Alicia Beltran, Salma M. Musaad, Tracey Dibbs, Anu Singh, Shria Chug, Amy Sisson, Anil Kumar Vadathya, Tom Baranowski, Teresia M. O’Connor

**Affiliations:** 1 USDA/ARS Children’s Nutrition Research Center, Baylor College of Medicine, Houston, Texas, United States of America; 2 Baylor College of Medicine, Houston, Texas, United States of America; 3 Rice University, Houston, Texas, United States of America; 4 The Texas Medical Center Library, Houston, Texas, United States of America; 5 Rice University, Electrical & Computer Engineering, Houston, Texas, United States of America; Ege University, Faculty of Medicine, TURKEY

## Abstract

**Objective:**

Accurate measurement of adult and child screen media use are needed to robustly assess its impact on health outcomes. Our objective was to systematically review screen media use measurement tools that have been validated against an objective “gold standard” tool.

**Methods:**

The search strategy was initially conducted in Medline Ovid and translated to Embase, Web of Science, PsychInfo and Cochrane. A modified natural language search was conducted in Google Scholar and IEEE. The initial search was conducted in March 2021, and an updated search was conducted in June 2022. Additional studies were included from the references. Studies had to describe the validation of a tool to measure screen media use on participants of any age against a ‘gold standard’ or comparable objective measure. The COnsensus-based Standards for the selection of health Measurement INstruments (COSMIN) was used to assess the criterion validity. Four authors reviewed the titles in two rounds and extracted data.

**Results:**

Twenty-nine articles were included in the review. Studies measured TV, computer, mobile device and social media site screen media use through: self or parent report, direct or video observation, computer and mobile device use tracking programs, and through other novel devices such as wearable devices and cameras. Correlations of self or parent report of screen media with the gold standard were lower than correlations of technology-based measures, and video observation with the gold standard. The COSMIN criterion validity ratings ranged from poor to excellent; most of the studies received a global score of fair or poor.

**Conclusions:**

Technology based validated tools that more directly measure screen use are emerging that have been validated against a gold standard for measuring screen use. However, practical, objective measures of diverse types of screen media use that have been tested on diverse populations are needed to better understand the impact of screen media use on the development and physical and mental health of children and adults.

## Introduction

Screen media devices such as desktop computers, laptops, tablets, cell phones, televisions, and gaming PCs have become widely accessible to children and adults. These electronic devices are commonplace for work in many industries, in schools for online learning environments, to communicate with others through texting, video calls, emailing and social media, and for multiple forms of entertainment, including watching movies and shows, playing videogames, and for content creation (e.g. videos for social media platforms) [1–4]. However, excessive use of digital devices may pose health and development risks to children and affect health outcomes in adults. Many children exceed recommended guidelines [3] and continue to spend more time on screens as they age. Children 0 to 8 years old spent an estimated two and a half hours daily using screens (mainly watching television or online videos on sites like YouTube and TikTok); tweens (8–12 year olds) spent more than five hours daily; and adolescents spent more than 8 hours daily [4]. One year old children reportedly spent nearly one hour daily using screens which increased to over two hours by the time they reach three years of age [5]. Among children and adults, screen media use has been associated with shorter sleep duration, less healthful diet, increases in body mass index, metabolic syndrome, lower physical activity levels, musculoskeletal pain, and negative mental health outcomes [6–13]. Alternatively, other studies using similar self-report measures showed positive outcomes on physiological indicators of inhibitory control and working memory [14]. Thus, screen media use has been extensive across all ages, but differences have been reported on the extent to which it contributes to health or cognitive functioning. Differences could be due to the qualities of the instrument used to measure screen use. Most health outcomes studies completed to date have used unvalidated tools for measuring screen use. The potential health implications from screen media use on development, as well as mental and physical health, and cognitive functioning provide a strong rationale for improving tools for measuring screen media use to ensure its accurate assessment.

Multiple methods for assessing screen use have been reported, including self-reported (e.g., questionnaires, television diaries, ecological momentary assessments, retrospective recall interviews); observations (e.g., direct observation, coded raw video or photo observation) and technology-based (e.g. mobile device tracking applications, computer software programs, television monitors) [15]. Important challenges in studying the effects of screen media use have been accurately measuring duration and type of device used [15]. Parent-proxy or child-self report of screen media use was identified as the most common approach employed in research on the impacts of screen media use among children, and only 11% of survey-based tools had psychometric validity published [16, 17]. Self-report methods are subjective and introduce biases and inaccuracies due to recall errors and errors of judgment, especially for children. Parent proxy reporting is similarly flawed [18]. For example, parents overestimated their child’s television time compared to an objective measure by 4 hours/week when no TV was present in the bedroom; but underestimated TV time by over 3 hours/week when the child had a television in their bedroom [19]. When comparing parent report of young children’s mobile device use with applications that track mobile device use, parents’ estimates were inaccurate, with 34.8% over-estimating and 35.7% underestimating their child’s device use [20]. To add to the confusion, many researchers have confounded sedentary time with screen media use by only using accelerometers to measure screen use behaviors [21], further limiting the accuracy of estimates of screen media use. Thus, the most common methods for assessing screen media use, self or proxy reports, are known to incorporate substantial error.

A crucial step in identifying and assessing health outcomes from screen media use is accurately measuring when and for how long individuals engage in its use across multiple devices. This is especially important since screen media use may have a more adverse impact on health than overall sedentary time [22]. The validity of a screen media use tool can be assessed by comparing it to direct or recorded observations [23]. Other recent systematic reviews [16, 24–26] have reported on methods for measuring television viewing, mobile device usage and other screen media use, with a focus on self-report measures. The current systematic review evaluates screen media use tools that have been validated against a “gold standard” or previously validated tool. The quality of the available research is assessed to identify gaps in knowledge and inform future research on the validity of screen media use tools.

## Methods

This systematic review followed the Preferred Reporting Items for Systematic Reviews and Meta-Analyses (PRISMA) protocol for reporting systematic reviews [27]. (See S1 File for the PRISMA checklist) The protocol was registered in the PROSPERO International register of systematic reviews CRD42021240268 [28].

A medical librarian (AS) developed a search strategy in Medline Ovid (see S1 Table) and then translated the strategy to the Embase, Web of Science, PsycINFO, and Cochrane databases. An initial search was conducted in March, 2021, which included titles published on and before 2021. A second search was conducted, which included titles published between March 2021 and June 2022. Search terms included the following MeSH descriptors as well as synonymous key words and phrases: screen time, television, motion pictures, video games, computers, cell phones, smartphones, internet, social media, computer-assisted instruction, time factors, behavior observation techniques, surveys and questionnaires, validation studies, and reproducibility of results. Additional modified natural language searching was conducted in Google Scholar and IEEE Xplore. The initial search produced 24,058 results, and the second search produced 4,183. Duplicates were removed after each search.

To be included in the review, studies must have described the validation of a tool to measure screen media use against a gold standard. Studies could include participants of any age, living in any part of the world. Studies had to be reported in the English language. No publication date limits were imposed. Reports from the gray literature (such as government reports, proceedings, dissertations, and theses) were not specifically searched. Articles were excluded if they did not report on the validation of a tool to measure screen media use. Studies focusing on phone calls and text message mobile device use only, but not mobile device screen time, were also excluded. The references of all included articles were further searched, and 6 additional articles were included from this source that met the inclusion/exclusion criteria and had not been found in the initial search.

Seven authors (AS, TD, AB, OP, SC, TG, OH), screened the titles and eliminated publications which clearly did not meet inclusion criteria. Each title was reviewed by at least two authors independently. Secondly, the remaining articles’ abstract and/or full text were reviewed by two authors independently to identify those which met the inclusion and exclusion criteria. Discrepancies about which articles to include or exclude were discussed with the entire research team until a consensus was reached.

Twenty-nine articles emerged which fit the inclusion and exclusion criteria. The data from the articles were extracted independently by authors working in pairs (TG, OH, AB, OP, SC) and results were compared by a third author for accuracy. Once the data abstraction was completed and compared, discrepancies were discussed by the research team until a consensus was reached. Data and data extraction forms were stored in a custom-made Access database.

Data extracted from each study included basic information such as sample size, year published, country where study was conducted, and sample characteristics. For study methods, the team extracted the media measured (television, desktop, laptop, gaming system, mobile phone, tablet, movie screens, e-readers, or other), measurement tool type being tested 1) self-reported or proxy-reputed assessment tool (media diary, recall interview, questionnaire, etc.), or 2) technology-based assessment tool (computer software, mobile device app, wearable cameras of devices, etc.); description of the objective measure used for comparison (direct observation, video observation, or other), setting (lab, in-home, public, or other), statistical metrics conducted, and a description of the research question. Extracted information about each study included age group (adults, children, unknown), and additional characteristics within age groups (preschool children, students, office workers). Race and ethnicity were extracted when available. In the case of studies which included multiple objectives or subsamples, data were extracted only from the population in which screen media use was measured and validated.

The methodological quality of studies was assessed using the COnsensus-based Standards for the selection of health Measurement INstruments (COSMIN) checklist specific for assessing criterion validity of studies [29]. COSMIN was designed to calculate a quality score for measurement properties. The items assessed by COSMIN were: 1) are missing items reported, 2) is there a description of how missing items were handled, 3) is the sample size adequate, 4) is the criterion a reasonable ‘gold standard’, 5) are there important flaws in the study, 6) do they report correlations or Area Under the Receive Operating Characteristic (ROC) Curve (AUC), and/or 7) is sensitivity and specificity determined. Four authors (AB, OP, TG, and OH) independently assigned a COSMIN rating of poor to excellent for each item per study. The agreement between coders was substantial [30–32] with a weighted kappa for all coders combined of 0.66 (95% CI 0.57–0.75). After kappas were calculated, the four research staff discussed discrepancies and agreed upon a final COSMIN rating for each item. According to a COSMIN scoring convention, the quality score per measurement property was obtained by taking the lowest rating of any item, or ‘worst score counts’ [29].

## Results

**Fig 1:**
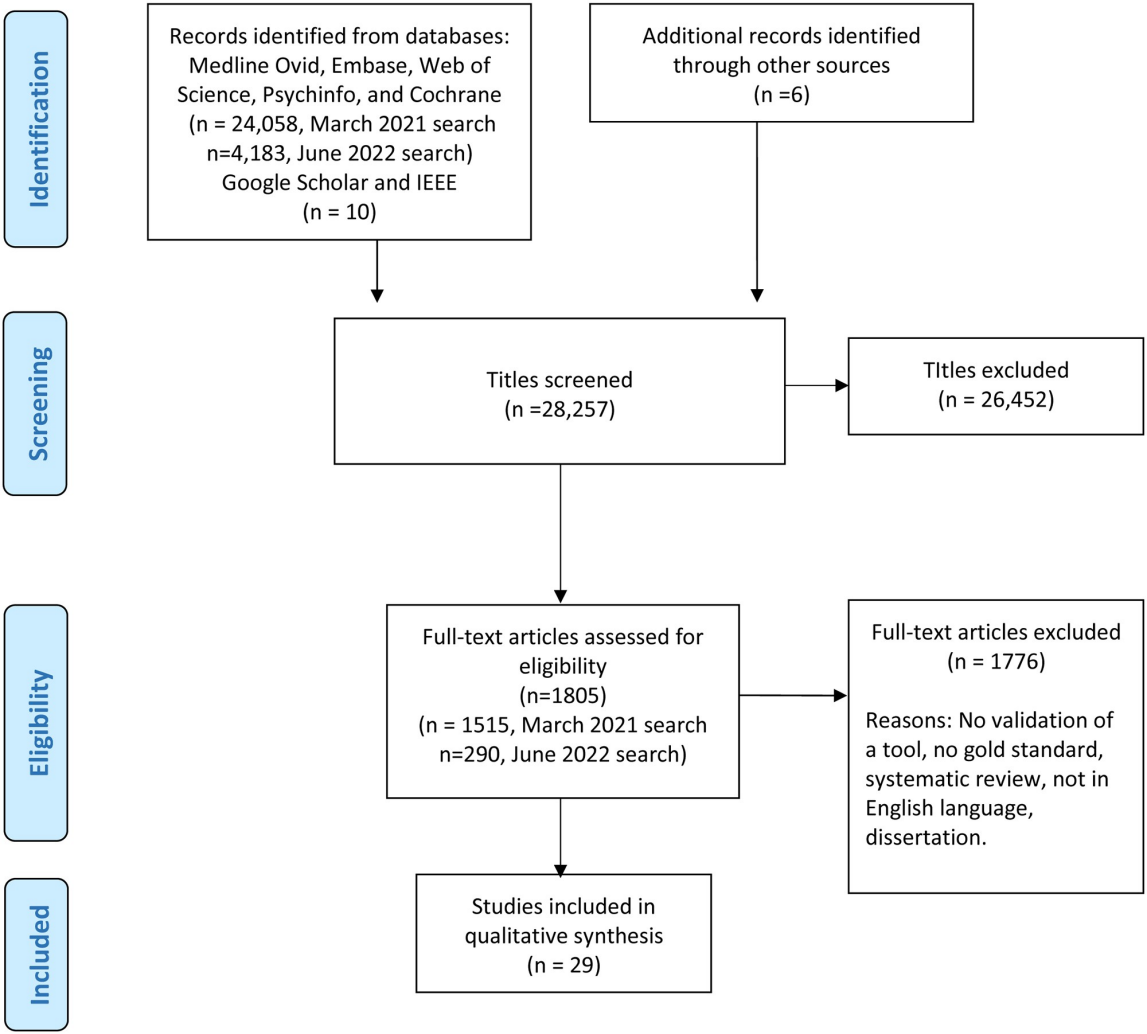
PRISMA flow diagram.

Following initial screening of 28,257 article titles, 26,452 were eliminated because they did not meet the inclusion criteria. The resulting 1,805 articles were assessed for eligibility by reviewing the abstract or full manuscript. Twenty-three articles met the criteria. Six additional articles emerged from the reference lists of relevant studies, and were also included, resulting in 29 articles that met inclusion criteria and which were included in the review (Fig 1). A description of the study participants of the 29 studies are summarized in Tables 1 and 2 describes the media measured, setting, measurement tools, and relevant statistical metrics of the included studies, organized by self-report or technology-based assessment tools. Sample sizes ranged from 2 to 1,211. The majority of studies were conducted in-Home (n = 9), followed by the workplace (n = 8), no location reported (n = 3), in a lab (n = 2), or in a combination of these locations (n = 7).

**Table 1:** Study participant characteristics.

Author (Year)	Target population	Country	Sample	Gender % Female	Race/Ethnicity n (%)
Anderson, D.R. et al. (1985) [23]	Preschool Children	USA	334	50.6	White 325 (97.3) African American 9 (2.7)
Mendoza, J.A., et al. (2013) [51]	Preschool Children	USA	96	42.7	Hispanic 96 (100)
Barr, R., et al. (2020) [53]	Preschool children (0–6 years)	USA	914	47	Not reported
Radesky, J.S., et al. (2020) [20]	Preschool Children	USA	346	48.8	White 258 (74.5) Other 86 (24.8) Not reported 2 (5.55)
Robinson, J.L., et al. (2006) [19]	Elementary-Age Children	USA	80	47.5	White 62 (77.5) African American 4 (5.0) Hispanic 7 (8.8) Multi-Racial 7 (8.8)
Vadathya, A. K., et al. (2022) [52]	Elementary-Age Children and preteens (?)	USA	42 (21 target children)	57	Non-Hispanic White 16 (38) Hispanic White 8 (19) Non-Hispanic Black 10 (24) Hispanic Black 2 (5) Asian 2 (5) Other (mixed or Hispanic Other 4 (10)
Wade, N., et al. (2021) [54]	Preteens aged 11–12	USA	67	46	White 23 (34) African American 5 (8) Asian 1 (2) Hispanic 20 (30) Other races: 18 (27)
Lee, P.H., et al. (2021) [59]	Adolescents and young adults (students)	China (Hong Kong)	187	72	Not reported
Verbeij, T., et al (2021) [55]	Adolescents	Netherlands	125	48	Not reported
Bechtel, R.B., et al. (1972) [56]	Adults and Children	USA	20	--	White 17 (85) African American 3 (15)
Junco, R. (2013) [34]	Adults (university students)	USA	45	73	White 40 (89) African American 2 (5) American Indian 1 (2) Asian 1 (2) Not reported 1 (2) (Hispanic 2 (5))*
Berolo, S., et al. (2015) [33]	Adults (university students/recent graduates)	Canada	47	40.5	Not reported
Geyer, K., et al. (2021) [48]	Adults (university students)	Spain	131	77.9	Not reported
Faucett, J. & Rempel, D. (1996) [42]	Adults (office workers)	USA	13 (subsample)	Not reported	Not reported
Homan, M.M. & Armstrong, T.J. (2003) [38]	Adults	USA	51	--	Not reported
Blangsted, A.K., et al. (2004) [44]	Adults (office workers)	Denmark	22	72.7	Not reported
Douwes, M., et al. (2007) [43]	Adults (office workers)	Netherlands	97	47	Not reported
Mikkelsen, S., et al. (2007) [46]	Adults (office workers)	Denmark	1211	73.6	Not reported
Chang, C.H., et al. (2008) [45]	Adults (office workers)	USA	20	65	Not reported
IJmker, S., et al. (2008) [47]	Adults (office workers)	Netherlands	572	48	Not reported
Yeh, J.Y., et al. (2009) [41]	Adults	Taiwan	24	83.3	Not reported
Otten, J.J., et al. (2010) [40]	Adults	USA	40	68	White 38 (95) Other, not reported 2 (5)
Araujo, T., et al. (2017) [36]	Adults	Netherlands	690	58	Not reported
Zhang, Y.C. & Rehg, J.M. (2018) [35]	Adults	USA	31	41.9	Not reported
Henderson, M., et al. (2021) [37]	Adults	USA	634	67.4	White 305 (48.11) Hispanic 177 (27.92) African American 138 (21.77) Other races: 14 (2.21)
Ohme, J., et al. (2021) [39]	Adults	Netherlands	404	55	Not reported
Trabulsi J., et al. (2021) [49]	Adults	USA	156	48.1	Not reported, described as “mix of ethnicities”
Kristensen P L., et al., (2022) [50]	Adults	Denmark	34	38.2	Not reported
Fletcher, R.R., et al. (2016) [58]	Not reported	USA	2	--	Not reported

*Race and ethnicity described separately.

Note: Table 1 is organized by the target population (youngest to oldest), then chronologically, then alphabetically.

**Table 2:** Screen media use assessment tools validated against gold standard, by type of assessment.

Reference	Media format	Setting	Measure	Type of Comparison	Measured content of screen media use	Duration of measurement	Statistical metric
**Self-report or proxy-report assessments**							
Bechtel, R.B., et al. (1972) [56]	TV	In-Home	3 TV viewing habits self-report viewing time questionnaire TV-diary (Parent-reported)	In home video recording observation of TV viewing	No	6-days	43.6% total combined mean agreement between questionnaire and video observation of viewing time in general
Anderson, D.R. et al. (1985) [23]	TV	In-Home	TV diary (Parent-reported) Direct Estimate (Parent reported global estimate of TV viewing)	In home video recording observation of TV viewing	No	Two 10-day periods	r = 0.84 (p < 0.001) between TV diary and video observation r = 0.27 (p < 0.01) between Direct Estimate and observation
Robinson, J.L., et al. (2006) [19]	TV	In-Home	Estimate of usual number of hours per week child spends viewing TV and computer screen (Parent-reported)	Electronic TV monitor (*TV Allowance*^™^)	No	3-weeks	r = 0.49 (p < 0.001) between parental estimate and TV monitor
Otten, J.J., et al. (2010) [40]	TV	In-Home	One item self-report screen time	Electronic TV monitor	No	3-weeks	r = 0.54 (p < 0.001) between self-report and TV monitor Agreement using Bland-Altman plot (95% of values fall within 2 SDs of the mean)
Faucett, J. & Rempel, D. (1996) [42]	Computer	Workplace	Self-report questionnaire of video display terminal use	Direct observation	No	3–5 hours daily for 4 days	r = 0.50 (p = 0.08), between self-report and direct observation of video display terminal use.
Mikkelsen, S., et al. (2007) [46]	Computer	Workplace	Self-report questionnaire	Computer use monitoring software (*WorkPace*)	No	4-weeks	r = 0.51, between self-report questionnaire and computer monitoring software of computer use.
IJmker, S., et al. (2008) [47]	Computer	Workplace	Self-report questionnaire	Computer use monitoring software (*WorkPace*)	No	14-days	18%r agreement (95% CI 15–21) between self-report and computer monitoring software of total computer time.
Araujo, T., et al. (2017) [36]	Computer, Laptop, Tablet	In-Home	Self-report questionnaire	Internet use tracking software	No	1-month	r = 0.294 (p < 0.01), between self-report of internet use *yesterday* and corresponding internet use duration using tracking software, r = 0.291 (p < 0.01), between self-report of internet use *on an average day* and corresponding internet use duration using tracking software.
Berolo, S., et al. (2015) [33]	Mobile phone	In-Home	Self-report questionnaire	Phone use tracking app (custom made)	Yes	1-week	Bland-Altman plots (difference between self-report and tracking app lies within 2 SD from the mean and is more variable with increasing minutes of use); Self report of typical phone use is 1.75 to 3.16 times higher than phone tracking app.
Barr, R., et al. (2020) [53]	TV, mobile phone, tablet	Not reported	Media diary Self-report questionnaire	Phone use tracking app (*Chronicle*)	No (Discussed but not measured)	Not reported	Kendall’s tau-b rank correlation coefficient was used between the category of parent-reported mobile device and the Chronicle output. t(35) = 0.41 (p < 0.001) for weekdays. t(33) = 0.20 (p < 0.11) for weekends. t(35) = 0.81 (p < 0.0001) when combining weekend and weekday time.
Radesky, J.S., et al. (2020) [20]	Mobile phone, Tablet	In-Home	Weekly estimate of child screen time	Screenshots of smartphone screen time on iOS devices, Phone use tracking app on Android devices (*Chronicle*)	Yes	7–10 days	31.3 percent agreement between parent self-report of child’s device use and iOS screen time screenshots 25.7 percent agreement between parent self-report and phone use tracking app
Geyer, K., et al. (2021) [48]	Mobile phone	Not reported	Self-report questionnaire	Phone use tracking app (custom made)	Yes	5-days	r = 0.19 (p = 0.025), between self-report of screen time and phone tracking app
Lee, P.H., et al. (2021) [59]	Mobile phone	In-Home, Public	Self-report questionnaire	Phone use tracking app (custom made)	Yes	7-days	r = -0.1 (p = 0.18), between self-report phone use and phone tracking app r = 0.21 (p = 0.005), between self-report time spent on social networking and phone tracking app r = 0.27 (p < 0.001), between self-report time on instant messaging, and phone tracking app r = 0.64 (p < 0.001), between self-report time spent on games and phone tracking app
Ohme, J., et al. (2021) [39]	Mobile phone	In-Home, Public	Self-report questionnaire	Screenshots of smartphone screen time on iOS devices	No (plan to measure in the future)	7-days	r = 0.3711 (p <0.05), between self-report of screen time and screen time screenshots.
Wade, N., et al. (2021) [54]	Mobile phone	Not reported	Self-report questionnaire Parent-proxy report questionnaire	Phone use tracking app *(Effortless Assessment of Risk States (EARS))*	Yes	4-weeks	r = 0.49 (p < .001) between self-report of screen time and phone tracking app. r = .1 (p = .43) between parent-proxy report of screen time and phone tracking app.
Junco, R. (2013) [34]	Social media time (Face-book)	In-Home, Public	Self-report questionnaire	Computer use monitoring software (time on *Facebook* and other computer use)	Yes	1-month	r = 0.587 (p < 0.001), between self-report time spent on Facebook and computer monitoring software r = 0.866 (p < 0.001), between self-report time spent on Twitter and computer monitoring software r = 0.628 (p < 0.001), between self-report time spent on email and computer monitoring software r = 0.335 (p < 0.05), between self-report time spent on a search engine, and computer use monitoring software
Henderson, M., et al. (2021) [37]	Social media time (Twitter)	In-Home	Self-report *Twitter* use questionnaire	Download of *Twitter* account data	Yes	1–114 months	r = 0.00–0.24, between self-reported time spent on *Twitter* and downloaded *Twitter* account data (Tweeting, retweeting, sharing photos/videos, direct messages)
Verbeij, T., et al. (2021) [55]	Social media	In-Home	Self-report questionnaire	Phone use tracking app (*Ethica App Usage Stream*) Experience Sampling Methodology (ESM)	Yes	3-weeks	r = 0.59 (< .001) between self-report via retrospective survey of total social media use on a typical week and phone tracking app r = 0.65 (< .001) between self-report via retrospective survey of total social media use on previous week and phone tracking app r = 0.55 (< .001) between ESM of total social media use and phone tracking app
**Technology-based Assessments**							
Vadathya A. K., et al., (2022) [52]	TV	In-Home, Lab	Television viewing detecting device (Family Level Assessment of Screen Use in the Home-Television (FLASH-TV))	Lab video recording observation	No	Approximately 90 minutes	Intraclass correlation (ICC): 0.725 between overall television viewing time estimated by automated device and video observation.
Fletcher, R.R., et al. (2016) [58]	TV, Computer, Laptop	Lab	Wearable wrist band color sensor with accelerometer	Lab video recording observation	No	--	AUC = 0.90 between wearable sensor and detection of TV screen AUC = .89 between wearable sensor and detection of computer screen AUC = 0.83 between wearable sensor and combined model of TV and computer screen detection
Zhang, Y.C. & Rehg, J.M. (2018) [35]	TV, Computer, Laptop, mobile phone, Tablet, Movie screen, e-reader	In-Home, Lab	Head-mounted wearable camera	TV detector, Eye tracking glasses, Video recording observation	No (plan to measure in the future)	Aware Home study (TV watching behavior in a living room environment) Twelve 1–2 minute sessions Multi-screen study (Attention to multiple types of screens using ground truth derived from eye tracking): 30minutes- 1hr—total of 13 hours Naturalistic study (Natural home environments): 1.87 hours to 39.82 hours	Precision: 0.917, of model identifying attention to screens Recall: 0.945 of model identifying attention to screens Aware Home study: AUC = 0.96 Multi-screen study: AUC = 0.98. Naturalistic study: AUC = 0.85.
Blangsted, A.K., et al. (2004) [44]	Computer	Workplace	Computer use monitoring software (*WorkPace*)	Video recording observation of two observers.	No	1 hour	r = 0.94 (p = 0.386) r = 0.93 (p = 0.005), between computer monitoring software and video recording observation of observer 1 and 2, respectively.
Chang, C.H., et al. (2008) [45]	Computer, Laptop	Workplace	Computer use monitoring software	Video recording observation	No	4 hours	r = 0.87–0.92 (p < 0.05), between computer monitoring software and video recording at 28-60second observation cutoff points of computer use.
Trabulsi J., et al. (2021) [49]	Mobile phone	Lab	Eye tracking glasses (Tobii Pro Glasses)	Human coded data of video observation of mobile phone use (Raw60)	Yes	Average of 5-minute sessions	R^2^ = 0.972 between human coded video data (Raw60) and NoMerge_NoDiscard eye tracking filter with velocity thresholds 7 and 10°/s R^2^ = 0.975 between human coded video data (Raw60) and NoMerge_NoDiscard eye tracking filter with velocity threshold 6°/s
Kristensen P L., et al., (2022) [50]	Mobile phone	In-home, Public	Phone use tracking app (*SDU DeviceTracker*)	iOS Apple Screen Time application Android ActionDash application	No	6–18 days	r = 0.88 (CI 0.84–0.91) between phone tracking app and Apple Screen Time application r = 0.99 (CI 0.98–0.99) between phone tracking app and Android ActionDash application
Homan, M.M. & Armstrong, T.J. (2003) [38]	Computer	Workplace	Self-report questionnaire Electronic activity monitoring (custom made)	Video recording observation	No	3-days	r = 0.78 (p < 0.01), between self-report and electronic activity monitoring of keying time on the computer r = 0.93 (p < 0.01), between electronic activity monitoring and video recording observation (work sampling) of keying time on the computer
Douwes, M., et al. (2007) [43]	Computer	Workplace	Self-report questionnaire Computer monitoring software	Direct observation, Computer use monitoring software (*WorkPace*)	No	2.5 hours	r = 0.41 (p = 0.001), between self-report and direct observation of computer use r = 0.86 (p = 0.001), between direct observation and computer use monitoring software
Yeh, J.Y., et al. (2009) [41]	Computer	Workplace	Self-report questionnaire Computer monitoring software	Video recording observation, computer use monitoring software (*Kblog*)	No	1 hour	r = 0.387 (p < 0.1), between self-report questionnaire and video observation of total computer use time use r = 0.960 (p < 0.0001), between computer monitoring software and video observation of total computer use time
Mendoza, J.A., et al. (2013) [51]	TV	In-Home, School	TV diary (Parent-reported) Ecological Momentary Assessment (EMA) Accelerometer	Electronic TV monitor (*TV Allowance*^™^),	No	Two 7-day periods (time 1 and time 2), 3–4 weeks apart	r = 0.45 (p < 0.001) at time 1, 0.55 (p < 0.001) at time 2 between TV diary and TV monitor r = 0.47 (p < 0.001) at time 1,0.51 (p < 0.001) at time 2 between TV diary and EMA r = -0.18 (p = 0.08) at time 1,–0.04 (p = 0.73) at time 2 between TV diary and Accelerometer

Note: Table 2 is organized by media format measured, then chronologically, then alphabetically. The results are sorted into three groups according to the type of measures compared: self-report, technology-based, or a combination of the two.

### Diversity of participants

Eighteen studies measured screen media use among adults [33–50], which included university students, office workers, and the general public. Seven studies [19, 20, 23, 51–54] measured screen media use among preschool, elementary school or preteen children. One [55] measured screen media use among adolescents. Two studies [56, 57] measured screen media use among more than one age group: Lee [57] measured mobile phone use among adolescents and young adults, and Bechtel [56] measured TV viewing time among adults and children. One study [58] did not report the ages of their study participants.

Most studies (n = 17) were conducted in the United States, followed by studies from the Netherlands (n = 5), Denmark (n = 3), Spain (n = 1), China (n = 1), Taiwan (n = 1), and Canada (n = 1). Ten studies included information about the racial or ethnic profile of their sample [19, 20, 23, 34, 37, 40, 51, 52, 54, 56]. Those reporting ethnicity showed most participants were White (Table 1).

### Variety of screens measured

Most studies (n = 18) assessed a self-reported or proxy-reported measurement tool against a gold standard, while seven studies reported on a technology-based tool and four studies assessed both a self-report and a technology-based tool (Table 2). Most studies also measured a single type of ‘screen’ in their study design. Six studies [19, 23, 40, 52, 53, 56] measured TV viewing exclusively; of those five were self-reported [19, 23, 40, 53, 56] and one was technology-based [52]. Nine studies [36, 38, 41–47] assessed measures of screen media use on a computer, four were self-reports [36, 42, 46, 47], two were technology-based [44, 45], and three tested both self-report and technology-based approaches [38, 41, 43]. In addition to measuring time spent in front of a computer screen, several studies also measured time spent using the keyboard and mouse. Nine studies [20, 33, 39, 48–50, 53, 54, 57] focused on mobile devices including phones and tablets, of which seven assessed self-report tools [20, 33, 39, 48, 53, 54, 59] and two tested technology-based tools [49, 50]. Three studies [34, 37, 55], using self-report approaches, looked at screen media use on specific social media sites like Facebook and Twitter on a computer screen. Four studies [35, 36, 53, 58] measured more than one screen including TVs, computers, laptops, and mobile phones and tablets, with two testing self-report [35, 53] and two testing technology-based assessments [35, 58].

### Correspondence between screen media use measurement and a gold standard

#### Television screens

Two studies compared a self-report with direct or video observation of TV viewing during the same time frame [23, 56]. Anderson et al. [23] found a correlation of 0.84 between the TV diary completed by parents and observation of child TV viewing using video recordings, and a correlation of 0.27 between a global estimate of TV viewing completed by the parent and video observation. Bechtel et al. [56] reported a 43.6% agreement between TV viewing questionnaire and staff coded video observations of TV viewing. Three studies compared self-report measures to a TV monitor such as TV Allowance^™^ (Family Safe Media, Park City, UT) as an objective measure of TV screen media use [19, 40, 51]. TV Allowance^™^ was a device which connected to a television or computer power cord and detected when the device was turned on (but not if someone was watching). It did not work on all TVs and is no longer available for purchase [60]. Robinson et al. [19] reported a correlation of 0.49 between parental report of child’s weekly TV viewing and the TV monitor. Otten et al. [40] found a correlation of 0.54 between a one item adult report of screen media use and the TV monitor installed in the participant’s homes. Mendoza et al. [51] reported correlations between 0.45–0.55 between child’s TV viewing duration as reported in a TV diary by parents versus the duration reported by the electronic TV monitor and correlations of 0.47–0.51 between the TV diary and an Ecological Momentary Assessment (EMA) approach, which prompted participants to answer brief surveys throughout the day about the child’s current behavior (i.e. “what activity is your child doing now?”). In addition, Mendoza et al. [51] used accelerometers to measure the child’s sedentary time and found correlations of -0.04–0.18 between the TV diary and sedentary time.

Only one study tested a technology-based assessment for measuring TV viewing compared to a gold standard. Vadathya et al. [52] used an automated device (*FLASH-TV*) to measure TV viewing of a target child. The device detected the presence of the target child in the room, differentiated their face from other persons in the room, and detected when the child was gazing at the TV using machine learning processing of video data captured in front of the TV. They found an Intraclass correlation (ICC) of 0.725 between the FLASH-TV estimation of the target child’s TV viewing duration and staff coding of the video records.

#### Computer screens

Four studies tested self-reported assessments of computer use to a gold standard, including direct observations, a computer use monitoring software, *WorkPace* (Niche Software Ltd., New Zealand), and internet use tracking software. Three studies exclusively measured self-report questionnaires against computer monitoring software [36, 46, 47] and one [42] compared self-report with video observations of participants’ computer use. Studies which used self-report measures had weak correlations to the gold standards [61]. Faucett & Rempel [42] reported a correlation of 0.50 between self-report and direct observation of computer use. Mikkelsen et al. [46] found a correlation of 0.51 of their self-report questionnaire and the computer use monitoring software. IJmker et al. [47] reported an 18% agreement between self-report and computer use monitoring software; Araujo et al. [36] reported correlations of 0.29 (recall of computer use the day before) and 0.29 (recall of computer use on an average day) with their custom-made internet use tracking software which captured each URL (Uniform Resource Locator, a web page address) accessed by the participant, as well as time spent on each URL.

An approach tested by Homan & Armstrong [38] was a custom-made electronic activity monitoring device consisting of external microprocessors which sensed keyboard, mouse and computer use. Two studies compared the computer monitoring software [44, 45] with video observations of participants’ computer use with correlations ranging from r = 0.87–0.92, and 0.93–0.94, respectively.

Three studies [38, 41, 43] used a triangulated approach by comparing self-report, technology-based and direct observation measurements. The technology-based programs, *WorkPace* and KBlog, work similarly to the Homan & Armstrong approach [38] by recording dynamic mouse and keyboard use and estimated total computer use based on these indicators. These three studies found stronger correlations between the direct observation and the technology-based measures, than the self-report measures. Homan & Armstrong [38] found a correlation of 0.78 with self-report and 0.93 between their custom-made electronic activity monitoring and video observations of computer use. Similarly, Douwes et al. [43] found a correlation of 0.41 between self-report and direct observation of computer use, in contrast with a correlation of 0.86 between their computer use monitoring software and direct observation of computer use. Yeh et al. [41] found a correlation of 0.387 between self-report and video observations of computer use, and a correlation of 0.960 between the computer monitoring software and video observation of computer use for the same period.

#### Mobile device screens

Seven studies compared self- or proxy-report of mobile device use (smart phone or tablet) to a device-use tracking application (app), including custom-made [33, 48, 59] and commercially available tracking apps [20, 33, 48, 50, 53, 54, 59] such as *Chronicle* [20, 53], *SDU DeviceTracker* [50], and *Effortless Assessment of Risk States* (EARS) [54]. Phone tracking apps are similar, in that they collect usage data from the operating system of Android mobile devices (phones and tablets) and record the name of the app used and duration of usage. Researchers can collect a report of the apps used on the device for the duration of the study. Barr et al. [53] compared parent-reported media diaries and reports of child’s mobile device use with the Chronicle app and found Kendall’s tau-b rank correlations ranging from 0.41–0.81. Berolo et al. [33] presented Bland-Altman plots showing that self-report of mobile screen media use was 1.75 to 3.16 times higher than their tracking app. Radesky et al. [20], reported 25.7% agreement between parent report and the *Chronicle* tracking app. Lee et al. [59] found a weak correlation of -0.1 between self-report of overall mobile screen use and the tracking app (See Table 2 for additional correlations found for categories of phone use). Geyer reported a correlation of 0.19 between their custom-made mobile use tracking app and self-report [48]. Wade et al. [54] reported correlations ranging from .1-.49 between the *EARS* app and self, or parent report of mobile device use.

Apple mobile devices, which function with the iOS operating system, have restrictions that currently prevent similar usage tracking apps to measure device use. To circumvent the restrictions, Ohme et al. [39] and Radesky et al. [20] asked participants with Apple devices to provide periodic screenshots of their Apple Screen Time application, which provides a report of the device’s usage. In addition to screenshots of iOS devices, when comparing parents’ weekly estimates of their child’s screen media use, Radesky et al. [20] found 31.3% agreement between the parent report and the iOS screenshots. Ohme et al. [39] exclusively compared mobile screen media use self-report questionnaire (on adults) and iOS screen time screenshots and found a correlation of 0.3711 between the two.

Two studies assessed technology-based approaches for measuring mobile device use to a gold standard. Kristensen et al. [50], designed a custom-made tracking app (SDU DeviceTracker) which is able to collect mobile use tracking data on iOS and Android devices. They found a correlation of 0.99 between their tracking app and Android’s built-in ActionDash application. In contrast, they found a lower correlation of 0.88 between their tracking app and Apple Screen Time application. Trabulsi et al. [49] compared the performance of eye tracking glasses, *Tobii Pro Glasses*, versus human coded video data of the participants’ eye movements using specific eye-tracking filters, or algorithms. The highest correlations they found were 0.972–0.975, between their *NoMerge_NoDiscard* (see Table 3 for specific distinctions between the filters used) filter and human coded video data.

**Table 3:** Consensus-based standards for the selection of health measurement instruments (COSMIN) checklist.

Author (Year)	1 Percentage of missing items	2 Missing items handled	3 Adequate sample size	4 Reasonable gold standard	5 Flaws in design or methods	6 Correlation or AUC reported	7 Sensitivity and specificity reported	Final Global score
Anderson, D.R. et al. (1985) [23]	Excellent	Excellent	Excellent	Excellent	Excellent	Excellent	N/A	**Excellent**
Mikkelsen, S. et al.(2007) [46]	Excellent	Excellent	Excellent	Excellent	Excellent	Excellent	NA	**Excellent**
IJmker, S., et al. (2008) [47]	Excellent	Excellent	Excellent	Excellent	Excellent	N/A	Excellent	**Excellent**
Trabulsi J., et al. (2021) [49]	Excellent	Excellent	Excellent	Excellent	Excellent	Excellent	N/A	**Excellent**
Homan, M.M., & Armstrong, T.J. (2003) [38]	Good	Good	Good	Excellent	Excellent	Excellent	N/A	**Good**
Douwes, M., et al. (2007) [43]	Excellent	Good	Good	Excellent	Excellent	Excellent	N/A	**Good**
Ohme, J., et al. (2021) [39]	Excellent	Excellent	Excellent	Good	Excellent	Excellent	NA	**Good**
Wade, N., et al. (2021) [54]	Excellent	Excellent	Good	Good	Excellent	Excellent	N/A	**Good**
Robinson, J.L., et al. (2006) [19]	Excellent	Excellent	Good	Good	Fair	Excellent	N/A	**Fair**
Otten, J.J., et al. (2010) [40]	Good	Excellent	Fair	Good	Fair	Excellent	N/A	**Fair**
Junco, R. (2013) [34]	Excellent	Good	Fair	Excellent	Fair	Excellent	N/A	**Fair**
Mendoza, J.A., et al. (2013) [51]	Excellent	Excellent	Good	Good	Fair	Excellent	N/A	**Fair**
Araujo, T., et al. (2017) [36]	Good	Good	Excellent	Excellent	Fair	Excellent	NA	**Fair**
Barr, R., et al. (2020) [53]	Excellent	Good	Excellent	Excellent	Fair	Excellent	N/A	**Fair**
Henderson, M., et al. (2021) [37]	Excellent	Excellent	Excellent	Excellent	fair	Excellent	N/A	**Fair**
Lee, P.H., et al. (2021) [59]	Good	Good	Excellent	Good	Fair	Excellent	NA	**Fair**
Verbeij, T., et al (2021) [55]	Excellent	Fair	Excellent	Good	Excellent	Excellent	N/A	**Fair**
Geyer, K., et al. (2021) [48]	Excellent	Excellent	Excellent	Good	Fair	Excellent	N/A	**Fair**
Vadathya, A.K., et al. (2022) [52]	Excellent	Excellent	Fair	Excellent	Excellent	Excellent	Excellent	**Fair**
Kristensen P L., et al., (2022) [50]	Excellent	Excellent	Fair	Good	Fair	Excellent	N/A	**Fair**
Bechtel, R.B., et al. (1972) [56]	Excellent	Excellent	Poor	Excellent	Fair	Poor	N/A	**Poor**
Faucett, J. & Rempel, D. (1996) [42]	Excellent	Fair	Poor	Excellent	Fair	Excellent	N/A	**Poor**
Blangsted, A.K., et al. (2004) [44]	Good	Good	Poor	Excellent	Fair	Excellent	N/A	**Poor**
Chang, C.H., et al. (2008) [45]	Good	Good	Poor	Excellent	Fair	Excellent	N/A	**Poor**
Yeh, J.Y., et al. (2009) [41]	Good	Good	Poor	Excellent	Fair	Excellent	N/A	**Poor**
Berolo, S. et al. (2015) [33]	Excellent	Excellent	Fair	Excellent	Fair	Poor	N/A	**Poor**
Fletcher, R.R., et al. (2016) [58]	Good	Good	Poor	Excellent	Fair	Excellent	N/A	**Poor**
Zhang, Y.C. & Rehg, J.M. (2018) [35]	Good	Fair	Poor	Excellent	Fair	Excellent	N/A	**Poor**

Note: Studies grouped by Final Global Score, then listed chronologically within score group.

#### Social media use

Three studies focused on screen media use spent on specific social media [34, 37, 55]. Junco [34] utilized a computer use monitoring software to compare self-reported time spent on Facebook, Twitter, as well as time spent on email and searches. They found correlations of 0.587 between self-report of time spent on Facebook, and 0.866 time spent on Twitter against the actual usage measured by the software (see Table 2 for additional correlations of other types of computer use). Henderson et al. [37] utilized a Python script to access and download participants’ activity on Twitter and compare it to their self-reported usage. They found correlations of 0.00 to 0.24 between self-report of Twitter use and downloaded data. Verbeij et al. [55] measured adolescents’ time spent on various social media platforms by comparing self-report questionnaires, the *Ethica* mobile use tracking app, and Experience Sampling Methodology (ESM) which was comprised of periodic text messages to the participant’s mobile device asking about their current activity on social media. Verbeij et al. [55] found a correlation of 0.55 between ESM and the mobile tracking app; 0.59 between self-report of total social media use on a typical week and the mobile tracking app; and 0.65 between self-report of total social media use on the previous week and the mobile tracking app.

#### Tools for measuring multiple screen media platforms

Fletcher et al. [58] and Zhang & Rehg [35] used novel devices to measure screen media use on a variety of screens. The former tested an optical color sensor with accelerometer worn on the wrist. The sensor was tested while the participants performed various activities in front of a TV, computer or laptop screen, and under varied lighting conditions. Fletcher et al.’s [58] preliminary data suggest their machine learning algorithms can differentiate the type of screen to which the participant was exposed (TV, computer or laptop). They reported scores of AUC of 0.90 for detecting a TV screen, 0.89 for detecting a computer screen, 0.83 for detecting both screens are present near the device. Zhang & Regh [35] tested a head-mounted wearable camera compared to eye tracking glasses, TV detector and video observation. They used machine learning algorithms to analyze and classify the video recorded by the wearable camera to identify when the participant was watching TV, or a variety of screens. Zhang & Regh [35] reported precision (percentage of image frames correctly classified by their system as the participant was watching the screen compared to video observation of TV viewing) of 0.917, and recall (percentage of image frames correctly detected as participant watching the screen compared to video observation) of 0.945 of the head-mounted wearable camera in identifying the TV screen. Additionally, they reported an AUC of 0.98 between Precision and Recall detecting multiple screen viewing (see Table 3 for additional sub-study findings).

#### Content of screen media measured

The tools described by Berolo et al. [33], Radesky et al. [20], Geyer et al. [48], Lee et al. [59], Wade et al. [54], Junco [34], Henderson et al. [37], Verbeij et al. [55], and Trabulsi et al. [49] captured the content of the screen media being measured; for example, if the participant viewed entertainment, educational, or other content. Barr et al. [53], Ohme et al. [39] and Zhang & Rehg [35] reported the screen media tool they used was capable of measuring content, but the published study did not include that analysis. None of the other studies described in this review included a measure of the content of screen media use.

### Methodological quality

Table 3 reports the methodologic quality of the included studies. Four studies were rated as *Excellent* [23, 46, 47, 49], four as *Good* [38, 39, 43, 54], twelve as *Fair* [19, 34, 36, 37, 40, 48, 50–53, 55, 59], and nine studies had a global score of *Poor* [20, 33, 35, 41, 42, 44, 45, 56, 58]. The poor ratings were due to a small sample size (<30) [35, 41, 42, 44, 45, 56, 58] and/or not reporting correlations or AUC in their results [20, 33, 56]. As per COSMIN, item 5 (flaws in design or methods) articles received a rating of fair if they had minor methodological flaws in the design or execution of the study such as having a small sample size, not reporting correlations or AUC, or they described a flaw in the design of the questionnaires they used [36, 37].

## Discussion

This review identified and described tools that have been validated against gold standard assessments to measure screen media use. Our review is unique and builds upon existing literature by identifying novel technology-based methods that measure screen media use. Such approaches may be more objective than self-report approaches but are still relatively early in their testing and use. Technology-based measures of screen media use, including TV monitors; internet, computer and mobile use tracking software; wearable cameras; wearable devices, and image processing machine-learning approaches, had higher correspondence with screen media use detected by direct and video observation (ranging from 0.73 [52] to 0.99 [50]) than self-report questionnaires (ranging from 0.00 [37] to 0.84 [23]). Self- or proxy-report via TV and media diaries [23] tended to have better correspondence to gold standard (0.84 [23]) than short survey style estimates of screen media use (0.54 [40], 0.50 [42], 0.51 [46], 18% agreement [47], 0.291–0.294 [36]). This is consistent with the systematic review by Parry et al [26], which found studies comparing self-report such as questionnaires and global estimates to objective measures tended to report low correlations; except for TV diaries, which reported moderate correlations with video observations and electronic TV monitors. The current review adds to this by finding correlations between technology-based approaches for measuring screen use and direct or video observations were high to very high.

A previous systematic review by Byrne et al. [16], noted the methods for assessing television viewing time had not kept pace with current research interest. They were not able to find any objective, device-based method of assessing screen media use. The present review identified a number of emerging objective methods to measure screen media use on a diversity of screens. Three studies in this review (Zhang & Regh [35], Fletcher et al. [58] and Vadathya et al. [52]) relied on technology-based approaches to measuring screen media use in combination with machine learning technology. These innovative approaches could transform research methods for measuring screen use, however, the samples were small and the methods warrant further testing. Computer and internet use tracking software programs such as the ones used by Homan & Armstrong [38], and Mikkelsen et al. [46] used motion trackers to measure hand and arm movements while using the keyboard and mouse, summing them and equating these to total computer usage time. These studies came from the fields of ergonomics and occupational health, wherein the researchers were not interested in measuring screen media use, but in finding objective measures of overall computer use to establish a relationship with postural problems and musculoskeletal pain. Other studies relied on existing technologies such as electronic TV monitors which detected whether the TV was on, but not whether an individual was actually gazing at the TV [19, 51]. One study assessed the use of accelerometers to measure sedentary time, as a proxy for screen use [51]. Another study reviewed the use of accelerometry or heart rate monitoring as a proxy to measure of TV viewing time [62]. Both posed additional challenges as not all screen media use is sedentary (e.g. active video games) and not all sedentary behavior involves use of a screen (e.g. reading a book, working on a puzzle, sitting and talking with friends). Clear guidelines to separate screen use and sedentary behaviors during assessments are available from the Sedentary Behavioral Research Network [21].

Most of the studies in this review measured television, computer, or mobile devices separately, limiting the ability to assess a participant’s total screen media exposure across different platforms. While TV viewing is still common, particularly among children [8], screen media use behaviors are evolving. Adults are spending less time on traditional televisions and spending more time on mobile devices, viewing online television, and gaming on consoles [2]. The significant rise in mobile devices has created a new ubiquitous medium where individuals spend time viewing screens. Tracking apps which capture mobile device use have been used as an objective measure of mobile screen media use because they do not rely on participant recall. Rather, they ‘read’ the device’s usage log. However, no studies compared mobile device tracking apps with video or direct observations. Video or direct observation is the current gold standard to measure screen media use; but it is time intensive, invasive, and in the case of mobile devices, impractical. However, such tracking apps are limited in measuring a person’s device use when the device is shared by multiple people. Assessment of mobile device use among young children can be more challenging because they are more likely to share a device with a sibling or parents [20]. Nevertheless, this technology has opened a new area of study of when and for how long individuals, especially adolescents and adults, engage with their mobile devices. Despite this, self-report continues to be widely used to measure screen media use [16, 24], even though the validity is low.

Few studies reported on the race and ethnicity of their samples. Those that did, tended to include White adults from the United States and Europe. Including participants from diverse backgrounds, ages and physical appearance is relevant because some screen media use detection devices, (such as the one used by Zhang & Rehg [35] and Vadathya A K., et al., [52]) rely on image processing of the participant’s face and/or eyes using machine learning algorithms. Skin color and lighting can influence the ability to detect screen media gaze. Diversity of participants in training and testing such approaches reduces the likelihood that these measurement methods will contain inherent bias [63]. Some are considering this in their technology development pipeline [52]. Similarly, the wrist-worn detector used by Fletcher et al. [58] is affected by movement, and children may be more likely to be physically active during screen use than adults. Additionally, screen media use measurement tools that work on adults may not work on children due to different types of screen use or recognition patterns. Validation studies are needed among children as well as adults.

Most of the studies in this systematic review were scored as poor or fair, using the COSMIN checklist for criterion validity. The low ratings were mostly due to methodological flaws, or small, non-representative numbers of participants.

### Limitations

The search strategy may have missed relevant articles due to the large number of studies interested in measuring screen media use. For example, the search strategies captured public health, psychology, medical and some educational journals. The search also included a natural search in the IEEE engineering database to capture new technologies from the engineering side. However, other fields of study, such as communications, may not have adequately been covered, making it possible that we may have missed relevant articles. Additionally, the key words used may have missed some articles in the ever-expanding field of screen media use. The review was limited to English-language articles and those published in peer-reviewed journals; the gray literature was not searched, all of which limit the scope of the review.

## Conclusions

Practical self-report measures of screen media use tend to be inaccurate when compared to gold standard assessments of screen media use. Technology-based assessment tools for measuring screen use, such as tracking apps, cameras, light sensors and image processing machine learning algorithms, demonstrated much higher correlations to gold standard assessments, but many are still in developmental stages and need further validation before they can be deployed. Studies are needed on the development and validation of accurate, but simple to deploy, technology-based measures of screen media use, especially with diverse populations including children and racially and ethnically diverse samples. This may facilitate understanding the impact of screen media use on academic performance, physical and mental health, and development among children and adults from a variety of backgrounds, and may demonstrate different impacts from different screen media exposure. These studies need to integrate data from diverse screen platforms and account for the fluid nature of multiple screen media use by people, the content of what is viewed, and multi-tasking across screens.

## Supporting information

S1 File PRISMA checklist. (DOCX)

S1 Table Search strategy for Medline. S1 Table contains the search strategy developed by librarian and used in Medline Ovid database and subsequently translated to other databases.
(DOCX)

## Abbreviations

AUC: Area Under the Curve
COSMIN: COnsensus-based Standards for the selection of health Measurement Instruments
EMA: Ecological Momentary Assessment
ESM: Experience Sampling Methodology
PRISMA: Preferred Reporting Items for Systematic Reviews and Meta-Analyses
ROC: Receive Operating Characteristic
