# Supplementary material for: Validated assessment tools for screen media use: A systematic review
Source: PLoS One. 2023 Apr 13;18(4):e0283714. doi: 10.1371/journal.pone.0283714 (PMC10101444; doi:10.1371/journal.pone.0283714)
Supplement: S1 Table — contains the search strategy developed by librarian and used in Medline Ovid database and subsequently translated to other databases. (DOCX) [file pone.0283714.s002.docx]

**S2 Table: Search Strategy for Medline**

|  | MEDLINE OVID | March 2021 Search | Updated June 2022 Search |
| --- | --- | --- | --- |
| 1 | Screen Time/ | 485 | 946 |
| 2 | Television/ or Motion Pictures/ or Video Games/ or Microcomputers/ or Computers, Handheld/ or Cell phones/ or Smartphone/ or Internet/ or Social Media/ or Computer-Assisted Instruction/ or Education, Distance/ | 147,475 | 161,901 |
| 3 | Time Factors/ | 1,202,459 | 1,227,436 |
| 4 | (time or "use" or usage).ti,ab,kw. | 5,724,115 | 6,251,093 |
| 5 | 3 or 4 | 6,520,125 | 7,059,850 |
| 6 | 2 and 5 | 55,834 | 62,986 |
| 7 | ((screen or "digital medi*" or "electronic medi*" or television* or TV or TVs or "motion picture*" or movie* or film* or DVD or DVDs or bluray* or "blu-ray*" or streaming) adj3 (time or "use" or usage)).ti,ab,kw. | 11,968 | 13,942 |
| 8 | (("video gam*" or "online gam*" or "computer gam*" or "gam* system*" or MMO or MMOs or MMORPG or MMORPGs) adj3 (time or "use" or usage)).ti,ab,kw. | 816 | 936 |
| 9 | ((microcomputer* or computer* or desktop* or "desk-top*" or laptop* or "lap-top*" or "mobile device*" or tablet* or ipad* or ereader* or "e-reader*") adj3 (time or "use" or usage)).ti,ab,kw. | 16,327 | 17,272 |
| 10 | (("cell phone*" or smartphone* or "smart-phone*" or "mobile phone*") adj3 (time or "use" or usage)).ti,ab,kw. | 4,657 | 5,701 |
| 11 | ((internet* or web* or "social media*" or Twitter* or Facebook* or YouTube* or TikTok* or Instagram* or MySpace*) adj3 (time or "use" or usage)).ti,ab,kw. | 12,973 | 15,708 |
| 12 | (("computer-aided instruction*" or "computer-aided education*" or "distance education*" or "distance learning*" or "e-learning*" or "remote education*" or "remote learning*") adj3 (time or "use" or usage)).ti,ab,kw. | 320 | 422 |
| 13 | 1 or 6 or 7 or 8 or 9 or 10 or 11 or 12 | 85,831 | 96,889 |
| 14 | Behavior Observation Techniques/ | 395 | 464 |
| 15 | exp "Surveys and Questionnaires"/ | 1,075,267 | 1,172,137 |
| 16 | (measur* or calculat* or assess* or observ* or survey* or questionnaire* or tool* or instrument*).ti,ab,kw. | 9,771,253 | 10,633,833 |
| 17 | 14 or 15 or 16 | 10,099,133 | 10,979,224 |
| 18 | Validation Study/ or Validation Studies as Topic/ or "Reproducibility of Results"/ | 473,336 | 511,668 |
| 19 | (validat* or validit* or reproducib* or reliab* or accura*).ti,ab,kw. | 1,886,190 | 2,108,728 |
| 20 | 18 or 19 | 2,065,097 | 2,292,212 |
| 21 | 13 and 17 and 20 | 11,112 | 12,729 |
| 22 | limit 21 to English language | 10,732 | 12,326 |
